# WNT5A induces release of exosomes containing pro-angiogenic and immunosuppressive factors from malignant melanoma cells

**DOI:** 10.1186/1476-4598-13-88

**Published:** 2014-04-26

**Authors:** Elin J Ekström, Caroline Bergenfelz, Verena von Bülow, Filiz Serifler, Eric Carlemalm, Göran Jönsson, Tommy Andersson, Karin Leandersson

**Affiliations:** 1 Cell and Experimental Pathology, Department of Laboratory Medicine, Lund University, Clinical Research Centre, Skåne University Hospital, Malmö SE-20502, Sweden; 2 Center for Molecular Pathology, Department of Laboratory Medicine, Lund University, Skåne University Hospital, Malmö SE-20502, Sweden; 3 Lund University Bioimaging Center, Lund, Sweden; 4 Department of Oncology, Clinical Sciences, Lund University, Lund, Sweden

**Keywords:** WNT5A, Exosome, Malignant melanoma, IL-6, MMP2, VEGF

## Abstract

**Background:**

Wnt proteins are important for developmental processes and certain diseases. WNT5A is a non-canonical Wnt protein that previously has been shown to play a role in the progression of malignant melanoma. High expression of WNT5A in melanoma tumors correlates to formation of distant metastasis and poor prognosis. This has partly been described by the findings that WNT5A expression in melanoma cell lines increases migration and invasion.

**Methods:**

Malignant melanoma cell lines were treated with rWNT5A or WNT5A siRNA, and mRNA versus protein levels of soluble mediators were measured using RT-PCR, cytokine bead array and ELISA. The induced signaling pathways were analyzed using inhibitors, Rho-GTPase pull down assays and western blot. Ultracentrifugation and electron microscopy was used to analyze microvesicles. Gene expression microarray data obtained from primary malignant melanomas was used to verify our data.

**Results:**

We show that WNT5A signaling induces a Ca^2+^-dependent release of exosomes containing the immunomodulatory and pro-angiogenic proteins IL-6, VEGF and MMP2 in melanoma cells. The process was independent of the transcriptional machinery and depletion of WNT5A reduced the levels of the exosome-derived proteins. The WNT5A induced exosomal secretion was neither affected by Tetanus toxin nor Brefeldin A, but was blocked by the calcium chelator Bapta, inhibited by a dominant negative version of the small Rho-GTPase Cdc42 and was accompanied by cytoskeletal reorganization. Co-cultures of melanoma/endothelial cells showed that depletion of WNT5A in melanoma cells decreased endothelial cell branching, while stimulation of endothelial cells with isolated rWNT5A-induced melanoma exosomes increased endothelial cell branching in vitro*.* Finally, gene expression data analysis of primary malignant melanomas revealed a correlation between WNT5A expression and the angiogenesis marker ESAM.

**Conclusions:**

These data indicate that WNT5A has a broader function on tumor progression and metastatic spread than previously known; by inducing exosome-release of immunomodulatory and pro-angiogenic factors that enhance the immunosuppressive and angiogenic capacity of the tumors thus rendering them more aggressive and more prone to metastasize.

## Background

Wnt signaling proteins are a family of highly conserved proteins that are important during developmental processes. They have also been implicated in several diseases, such as cancer and diseases with an inflammatory component [1,2]. Canonical Wnt signaling, exemplified by WNT3A, is the most well characterized Wnt signaling pathway, leading to activation of β-catenin [1,3]. Non-canonical signaling via WNT5A can inhibit β-catenin signaling but also activate distinct signaling pathways, independent of β-catenin. Activation of non-canonical WNT5A signaling can lead to several different outcomes, such as activation of Ca^2+^ signaling, activation of small Rho-GTPases (Cdc42, Rac1 and RhoA), Calmodulin-Kinase II, PKC, PKA, and JNK [3-5].

Malignant melanoma is a highly aggressive cancer form which once spread, has a 5-year survival rate of 5% [6]. For the tumor to spread to distant sites, the formation of new vessels is required, a process known as angiogenesis. In malignant melanoma, angiogenesis is correlated to the transition of the tumor from the radial growth phase to the more invasive vertical growth phase [7]. Several secreted factors regulate angiogenesis such as VEGF, IL-6, Matrix metalloproteinase 2 (MMP2), IL-8 and FGF [8,9]. Some of these factors are also important in immunomodulation and the over-expression of these, by either the malignant melanoma cells or by infiltrating immune cells, can lead to enhanced metastasis due to induction of a local or systemic immunosuppression that is beneficial for the tumor cells escape from immune recognition and eradication [10]. The later stages of melanoma including the spread to distant sites and the formation of metastasis have been shown to be promoted by an increased non-canonical WNT5A signaling. In line with this, a high WNT5A expression was also correlated to a poor prognosis in melanoma patients [11]. This could partly be explained by the observations that; in vitro*,* WNT5A increases migration and invasion of malignant melanoma cells [12] and in vivo, WNT5A signaling increases the spread and tumor formation of lung metastasis [13].

Exocytosis, or cytokine secretion, is a process with important implications in most tissues and cellular systems. Despite being widely studied, there are still questions to be answered regarding the molecular mechanisms behind this process [14]. Briefly, activation of specific receptors causes an immediate release of preformed mediators from secretory granules. Regulated exocytosis pathways that are not constitutive in mode of action, are generally induced by an increased intracellular Ca^2+^-signal. This signal causes a complex reorganization of the filamentous actin (F-actin) that is facilitated by cellular mediators such as the small Rho GTPases Cdc42 and Rac1 and the Synaptic soluble NSF attachment protein receptors (SNAREs). Among these are the proteins syntaxins, Soluble NSF Attachment Proteins (SNAPs) and vesicle-associated membrane proteins (VAMPs). The VAMPs can be divided into tetanus neurotoxin (TeNT)-sensitive and -insensitive VAMPs [15]. Questions regarding the specific function and regulation of the actin cytoskeleton in secretory processes have been raised. However, an increase in intracellular calcium is necessary for cortical F-actin disassembly and its subsequent reorganization. Cdc42 and Rac1 have previously been shown to regulate the basolateral exocytosis and secretion of cytokines in polarized epithelial cells [16]. It was also shown that the polarization of cytolytic effectors in immune cells was regulated by Cdc42 [17].

Exosomes are 30–90 nm non-plasma membrane-derived vesicles that are formed in endosomal compartments called multivesicular endosomes and are released by a wide range of mammalian cells [18,19]. They contain various molecules ranging from endosomal markers (e.g. hsp70 and CD63) to signaling proteins (IL-6, IL-8, VEGF, Tissue inhibitor of metalloproteinases (TIMP-1/2) and FGFα) and mRNAs. The released exosomes merge with and empty their content into other cells, thus contributing to an intercellular communication. Tumor cells are known to have an exacerbated exosome secretion that has been linked to angiogenesis, metastatic spread and immunosuppression [18]. Exosome secretion can be constitutive or regulated by for instance growth factors. The molecular mechanism involves tetraspanins (e.g. CD63), activation of the Rab family of proteins and probably also certain SNAREs (e.g. Rab5b) [18-20]. Regulated exosome secretion can be Ca^2+^-induced and dependent on cytoskeletal reorganization [18,19]. It has previously been shown that the exosome dependent protein Rab35, can mediate the transport of Cdc42 and Rac1 to the plasma membrane to remodel the actin-structures [21]. We have previously shown that WNT5A induces an intracellular Ca^2+^-increase in human malignant melanoma and breast cancer cells [5,22]. We have also shown that WNT5A induced a specific activation of Cdc42 and to some extent Rac1 in human breast cells [5]. WNT5A has previously been shown to activate Cdc42 and induce cytoskeletal changes in fibroblasts [23].

Here we show, that malignant melanoma cell lines treated with recombinant (r)WNT5A induces a prominent, immediate release of immunomodulatory and pro-angiogenic factors IL-6, IL-8, VEGF and MMP2, while transcriptional activation of these genes remained unaffected. The release was inhibited by calcium chelation and expression of a dominant negative Cdc42. Neither Brefeldin A nor TeNT inhibited the WNT5A-induced release of the soluble mediators. Instead we show that WNT5A induces release of exosomes containing IL-6, IL-8, VEGF and MMP2. Using gene expression data of 223 primary malignant melanomas from the study by Harbst et al. [24], we further revealed a correlation between WNT5A expression and the angiogenesis marker ESAM. We also show that knock-down of WNT5A in malignant melanoma cells induced a decrease in endothelial cell branching in co-culture experiments with melanoma cells *in vitro*, suggesting that WNT5A might have an effect on tumor progression in malignant melanoma, through induction of a broad release of soluble mediators.

## Results

### WNT5A increases secretion of IL-6, IL-8 and VEGF in cell culture supernatants

**Figure 1:**
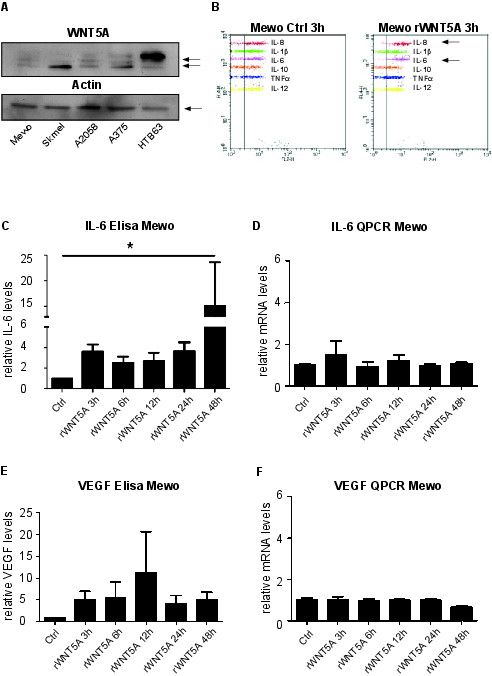
**WNT5A increases secretion of IL-6, IL-8 and VEGF in cell culture supernatants. (A)** Western blot of endogenous WNT5A protein expression in melanoma cell lines Mewo, SkMel28, A2058, A375 and HTB63. Anti-β-actin was used as loading control. Picture is representative of at least 3 experiments. **(B)** Mewo cells treated with carrier (0.1% BSA in PBS; Ctrl) or 0.2 μg/ml rWNT5A for 3 h. A set of inflammation cytokines were measured using BD Cytometric Bead Array (CBA) and both IL-6 and IL-8 were produced upon a short (3 h) rWNT5A stimulation. **(C)** IL-6 levels in Mewo cell culture supernatants were measured using IL-6 Elisa. Mewo cells were treated with 0.2 μg/ml rWNT5A or carrier for 3, 6, 12, 24 and 48 h and IL-6 in supernatants was measured. The bar graphs show average fold increase as compared to carrier (ctrl) for each time point indicated. The experiment was performed at least 4 times. Error bars represent SEM. **(D)** Cells from experiment 1C were used for IL-6 RT-QPCR. The experiment was performed at least 3 times. Error bars represent SD. **(E)** Supernatants from 1C were used to measure VEGF levels after WNT5A treatments. Error bars represent SEM. **(F)** Cells from 1C were used for VEGF RT-QPCR. The experiment was performed 3 times. Error bars represent SD. * = p <0.05 by ANOVA test.

It had previously been reported that WNT5A expression was connected to the presence of the immunomodulatory and pro-angiogenic factor IL-6 in supernatants from malignant melanoma cells [13]. However, little evidence exists when it comes to WNT5A induced gene expression of IL-6. We therefore decided to analyze this in more detail. We first determined the basal expression levels of WNT5A protein in 5 malignant melanoma cell lines, Mewo, SKmel28, A2058, A375 and HTB63 (also known as HT-144). Based on the low expression of WNT5A in Mewo and A375 cells and the high expression of WNT5A in HTB63 cells we decided to use these three cells lines in our further studies (Figure 1A). The Mewo cells are wild type for the V600E BRAF mutation and the A375 and HTB63 cell lines both carry this mutation [25,26]. We next treated Mewo cells with recombinant WNT5A (rWNT5A) for 3 h and measured a set of inflammatory cytokines. As shown in Figure 1B, this short incubation with rWNT5A induced a prominent secretion of both IL-6 and IL-8. We subsequently confirmed the WNT5A induced secretion of IL-6 using Elisa (Figure 1C). rWNT5A also induced IL-6 secretion over a longer time period as measured by Elisa at 3 h, 6 h, 12 h, 24 h and 48 h (Figure 1C). We have previously shown that WNT5A induces IL-10 [27] and IL-6 [28] in human monocytes. In Mewo cells however, IL-10 was not even expressed at the mRNA level (data not shown). The effect of IL-6 upon rWNT5A stimulation in Mewo cells was also investigated by RT-QPCR but there was no significant increase in IL-6 mRNA levels in Mewo cells (Figure 1D). This was also confirmed in A375 cells (Additional file 1: Figure S1 A-B) and A2058 cells (Additional file 1: Figure S1 C-D). PMA/ionomycin stimulation for 12 h did induce a prominent increase in IL-6 mRNA expression in Mewo cells showing that the Q-PCR was optimized (Additional file 1: Figure S1 E). IL-6 and VEGF have both been shown to increase the angiogenic potential of malignant melanoma. As shown in Figure 1E-F, treatment of Mewo cells with rWNT5A was surprisingly also found to increase secretion of VEGF in the cell culture supernatant without affecting the VEGF mRNA levels (Figure 1E-F). The same pattern, that rWNT5A increases VEGF secretion but not the mRNA expression, was also seen in the A375 cells (data not shown).

### WNT5A siRNA decreases secretion in cell culture supernatants

**Figure 2:**
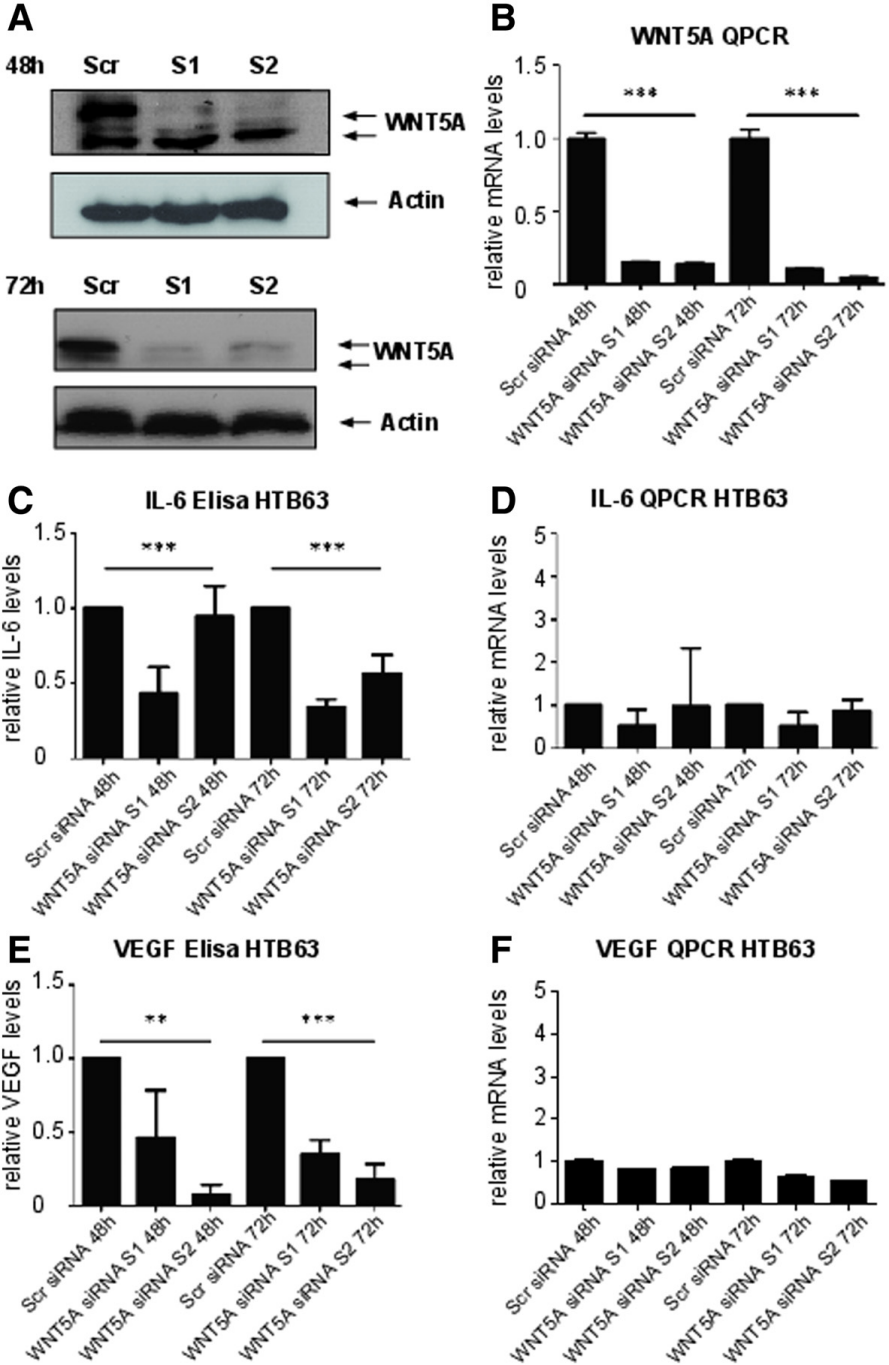
**WNT5A siRNA decreases IL-6 and VEGF secretion in cell culture supernatants.** The efficiency of WNT5A siRNA transfection was determined using Western blot **(A)** and RT-QPCR **(B)** 48 h and 72 h after transfection. Anti-β-actin was used as loading control. Cell culture supernatants were analyzed for differences in IL-6 protein **(C)** and VEGF protein using Elisa **(E)**. The experiment was performed at least 3 times. Error bars represent SEM. Differences in IL-6 **(D)** and VEGF **(F)** mRNA from the same experiments were analyzed using RT-QPCR. The experiment was performed at least 3 times. The bar graphs show average fold decrease as compared to scramble siRNA (ctrl) for each time point indicated. Error bars represent SD. *** = p <0.001, ** = p <0.01 by ANOVA test.

To further investigate the effects of WNT5A on production of IL-6 and VEGF in melanoma cells, siRNA was used to knock down the expression of WNT5A in HTB63 cells. The efficiency of the knockdown was determined at the mRNA level and protein level by Western blot and QPCR respectively (Figure 2A and B). After WNT5A knockdown, IL-6 secretion was reduced at 48 h and 72 h as measured by Elisa (Figure 2C). There was no difference in IL-6 expression on the mRNA levels neither at 48 h nor at 72 h (Figure 2D). Secreted VEGF levels were also reduced at 48 h and 72 h after siRNA transfection but there was no difference in mRNA levels after 48 h and 72 h (Figure 2E-F).

### WNT5A increases secretion by inducing exocytosis

**Figure 3:**
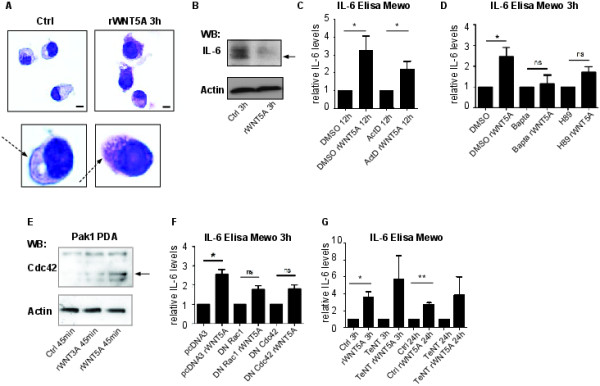
**WNT5A induces a Ca**^**2+**^**- and Cdc42-dependent release of soluble mediators. (A)** Mewo cells treated with rWNT5A for 3 h were paraffin embedded and stained with hematoxylin & eosin (HE). Scalebars = 10 μm. Arrows indicate differences in the cell cortex. **(B)** IL-6 Western blot (WB) detecting changes in intracellular IL-6 following cytokine release upon WNT5A treatment for 3 h. **(C)** Mewo cells treated with DMSO or Actinomycin D for 12 h together with rWnt5 or carrier. The bar graphs show average fold increase as compared to carrier (ctrl) for each time point and treatment indicated. Error bars represent SEM. n = 4. **(D)** Mewo cells were pre-treated with DMSO, the Ca^2+^-chelator Bapta-AM or PKA inhibitor H89 for 30 min prior to WNT5A treatment for 3 h. The bar graphs show average fold increase as compared to carrier (ctrl) for each time point and treatment indicated. Error bars represent SEM. n = 4. **(E)** Cdc42 is activated by WNT5A in Mewo cells. Western Blot of a GST-Pull down assay of active Cdc42, upon rWNT5A stimulation for 45 min. Controls are carrier (Ctrl) or rWNT3A for 45 min. **(F)** Transfection of Mewo cells with DN-Cdc42 or -Rac1 inhibits the WNT5A induced secretion of IL-6. Empty vector used as control. The data shown are ratios of rWNT5A treated (3 h)/untreated cells for each plasmid transfected and normalized against the empty vector control. n = 3. **(G)** Mewo cells were pre-treated with Tetanus toxin, TeNT, for 30 min prior to WNT5A treatment for 3 h or 24 h. The bar graphs show average fold increase as compared to carrier (ctrl) for each time point and treatment indicated. Error bars represent SD. n = 2. * = p <0.05 ** = p <0.01 by Student’s *t*-test. IL-6 levels in supernatants were detected using Elisa.

In order to investigate the mechanism behind the increased release of IL-6 and VEGF by WNT5A, Mewo cells were stimulated with rWNT5A for 3 h and subsequently paraffin embedded and stained with hematoxylin and eosin. A prominent cell border was detected in untreated cells and this was less pronounced in cells treated with WNT5A (Figure 3A). We then stained untreated cell or cells treated with WNT5A with phalloidin, to detect changes in the F-actin cytoskeleton, and detected a re-organization of F-actin following WNT5A treatment (Additional file 1: Figure S2 A). In untreated cells, the F-actin was primarily localized at the cell cortex whereas in WNT5A stimulated cells (3 h) the F-actin was primarily localized in the cytosol and less so in the cortical region. We next analyzed the effects of rWNT5A on the intracellular levels of IL-6 by Western blot and found that cell lysate from cells treated with WNT5A had less IL-6 than cell lysate from cells treated with carrier (Figure 3B). Furthermore, the rWNT5A-induced increase of IL-6 levels in Mewo supernatants, were not significantly affected by Actinomycin D (Figure 3C), a general inhibitor of transcription. The increases in IL-6 following WNT5A treatment could however, be inhibited by pre-treatment with the Ca^2+^ chelator Bapta and the PKA inhibitor H89 (Figure 3D).

### The WNT5A induced exocytosis is dependent on the small RhoGTPase Cdc42

It has previously been shown that the actin regulatory protein Cdc42, a small RhoGTPase, is activated by WNT5A in breast cells and also that it is important for exocytosis processes in different contexts [29-31]. It was furthermore shown that Cdc42 is important for VEGF-driven angiogenic effects in melanoma [32]. To investigate the involvement of Cdc42 in the present WNT5A induced effects, we first confirmed that rWNT5A indeed activated Cdc42 also in malignant melanoma Mewo cells (Figure 3E). Since we, and others have shown that WNT5A activates Cdc42 [5,23], we next analyzed whether expression of a dominant negative (DN) version of either Cdc42 or Rac1 affected the WNT5A induced exocytosis. We therefore transfected Mewo cells with DN-Cdc42 or DN-Rac1 prior to a 3 h rWNT5A stimuli and analyzed the secreted IL-6 and VEGF levels. As shown in Figure 3F, WNT5A induced IL-6 secretion was inhibited by both DN-Cdc42 and DN-Rac1. Also VEGF secretion was inhibited by DN-Cdc42 and slightly inhibited by DN-Rac1 (Additional file 1: Figure S2 B).

### The WNT5A induced cytokine release is not affected by TeNT

Cdc42 has previously been shown to activate distinct exocytosis processes and also to directly bind certain SNAREs [29-31]. As mentioned above, the VAMPs can be divided into TeNT-sensitive and -insensitive VAMPs. To investigate what molecular mechanism that was affected by WNT5A in more detail we therefore next performed a secretion experiment, using TeNT. Interestingly, TeNT did not inhibit the WNT5A induced exocytosis of IL-6 (Figure 3G) or VEGF (data not shown). Neither did Brefeldin A, a general inhibitor of the classical ER-Golgi secretory pathway (Additional file 1: Figure S2C).

### IL-6, VEGF and MMP2 are released upon freeze-thawing of supernatants

**Figure 4:**
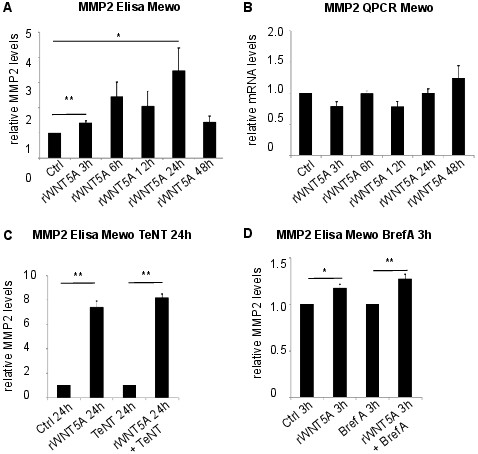
**WNT5A induces MMP2 release in Mewo cells. (A)** MMP2 levels in cell culture supernatants were measured using MMP2 Elisa. Mewo cells were treated with 0.2 μg/ml rWNT5A or carrier for 3, 6, 12, 24 and 48 h and IL-6 in supernatants was measured. The bar graphs show average fold increase as compared to carrier (ctrl) for each time point indicated. Error bars represent SEM. The experiment was performed at least 4 times. **(B)** Cells from experiment 4A were used for MMP2 RT-QPCR. Error bars represent SD. **(C)** Mewo cells were pre-treated with Tetanus toxin, TeNT, for 30 min prior to WNT5A treatment for 24 h. MMP2 levels in cell-culture supernatants were detected using Elisa. The bar graphs show average fold increase as compared to carrier (ctrl) for each time point and treatment indicated. Error bars represent SEM. n = 2. **(D)** Mewo cells were pre-treated with Brefeldin A, Bref A, for 30 min prior to WNT5A treatment for 3 h. MMP2 levels in cell-culture supernatants were detected using Elisa. The bar graphs show average fold increase as compared to carrier (ctrl) for each time point and treatment indicated. Error bars represent SEM. n = 4. * = p <0.05 ** = p <0.01 *** = p <0.001 by Student’s *t*-test.

We next chose to investigate the release of MMP2 since it is a protein that is involved in metastasis and also is secreted through the classical secretory pathway [33]. Secretion of MMPs is central to control degradation of extracellular matrix and invasion [34,35]. It has also been shown that small RhoGTPases promotes MMP2 secretion [36]. Also release of MMP2 was induced by a short stimulation of rWNT5A (3 h) an effect that lasted up to 24 h of rWNT5A stimulation (Figure 4A). Just as for IL-6 and VEGF, the mRNA levels of MMP2 remained unaffected (Figure 4B). We also evaluated whether DN-Cdc42 and -Rac1 would affect MMP2 release and as shown in Additional file 1: Figure S2D (left), overexpression of DN-Cdc42 inhibited the rWNT5A-induced release of MMP2 slightly and to a lesser extent also by DN-Rac1. Furthermore, neither TeNT nor Brefeldin A inhibited the rWNT5A induced release of MMP2 (Figure 4C-D and Additional file 1: Figure S2 D right) indicating that the mechanism behind the rWNT5A induced secretion of soluble mediators, did not act via the classical secretion pathway.

**Figure 5:**
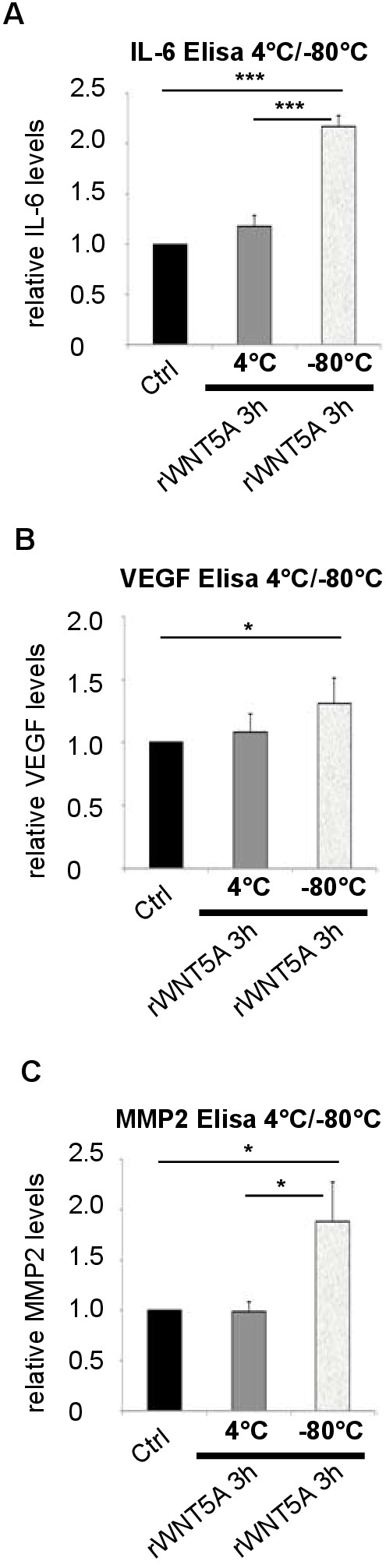
**Freeze/thawing releases the soluble mediators from the supernatants. (A)** IL-6 levels as measured by Elisa in cell culture supernatants of Mewo cells that were treated with 0.2 μg/ml rWNT5A or carrier for 3 h and subsequently was freshly run on Elisa (4°C) or freeze/thawed for two cycles -80°C/4°C before analyses. The bar graphs show average fold increase as compared to carrier (ctrl) for each treatment indicated. Error bars represent SD. n = 2. **(B)** VEGF levels as measured by Elisa in cell culture supernatants of Mewo cells that were treated with 0.2 μg/ml rWNT5A or carrier for 3 h and subsequently was freshly run on Elisa (4°C) or freeze/thawed for two cycles -80°C/4°C before analyses. The bar graphs show average fold increase as compared to carrier (ctrl) for each treatment indicated. Error bars represent SD. n = 2. **(C)** MMP2 levels as measured by Elisa in cell culture supernatants of Mewo cells that were treated with 0.2 μg/ml rWNT5A or carrier for 3 h and subsequently was freshly run on Elisa (4°C) or freeze/thawed for two cycles -80°C/4°C before analyses. The bar graphs show average fold increase as compared to carrier (ctrl) for each treatment indicated. Error bars represent SD. n = 2. * = p <0.05; *** = p <0.001 by Student’s *t*-test.

We next aimed to investigate a more precise mechanism behind the rWNT5A-induced secretion. In all initial experiments (Figures 1, 2, 3, 4), the Elisa’s were performed using previously frozen lysates. Therefore, we subsequently performed experiments using freshly prepared supernatants from rWNT5A stimulated Mewo cells (3 h) or supernatant that had gone through two cycles of freeze/thawing at -80°C/4°C. As shown in Figure 5, we could see that only supernatants from rWNT5A stimulated Mewo cells that had been frozen/thawed, showed an increased amount of soluble mediators IL-6 (Figure 5A), VEGF (Figure 5B) and MMP2 (Figure 5C). The actual concentrations measured are shown in Additional file 1: Figure S3 A.

### rWNT5A induces exosomes release containing the soluble mediators

**Figure 6:**
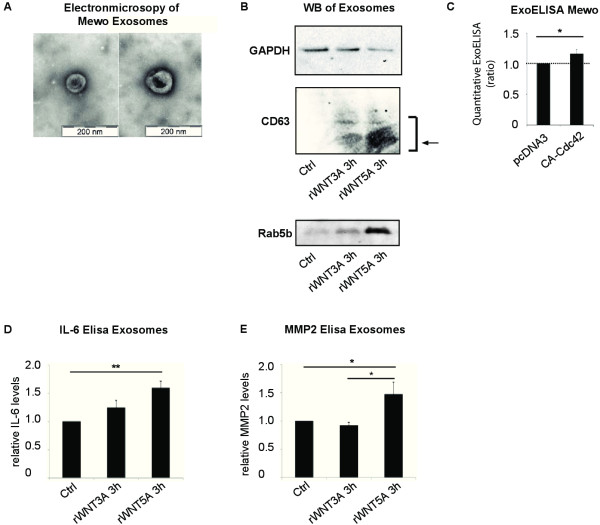
**WNT5A induces release of exosomes that contain solube mediators. (A)** Electronmicroscopic picture of isolated exosome fraction from supernatant of Mewo cells treated with 0.2 μg/ml rWNT5A for 3 h. **(B)** Western blot of isolated exosomes from supernatant of Mewo cell stimulated with 0.2 μg/ml rWNT5A, carrier or 0.1 ng/ml rWNT3A for 3 h. GAPDH (upper), CD63 (center) and Rab5b (lower). **(C)** Quantitative Exosome Elisa (ExoELISA) of isolated exosome fraction from Mewo cells transfected with pcDNA3 or CA-Cdc42. The bar graphs show the relative increase of exosomes produced in CA-Cdc42 transfected as compared to pcDNA3 transfected Mewo cells. Error bars represent SEM. n = 5. * = p <0.05 by Student’s *t*-test. **(D)** IL-6 or **(E)** MMP2 levels as measured by Elisa of frozen exosome fractions isolated from supernatant of Mewo cells that were treated with 0.2 μg/ml rWNT5A, carrier or 0.1 ng/ml rWNT3A for 3 h. The low protein yield in the isolated exosome fractions might explain the higher relative background level in the control as compared to IL-6 measured in supernatant. The bar graphs show average fold increase as compared to carrier (ctrl) for each treatment indicated. Error bars represent SD. n = 3. * = p <0.05, ** = p <0.01 by Student’s *t*-test.

One exocytosis mechanism that is known to affect the concomitant release of a wide variety of mediators is that of exosomes [19,37]. IL-6 mRNA transcript is induced in immune cells upon exosome stimulation. This was recently ascribed a mechanism involving TLR2 activation with subsequent IL-6 mRNA induction [38]. Since we did not observe changes in IL-6 mRNA levels we decided to analyze the TLR2 expression levels on the different malignant melanoma cell lines used and found that, compared to monocyte-derived myeloid dendritic cells (Mo-mDCs), the malignant melanoma cell lines (A375, Mewo and HTB63) lacked TLR2 expression (Additional file 1: Figure S3 B). With this, together with the observations described in Figure 5 in mind, we next evaluated whether rWNT5A could induce release of exosomes containing already pre-formed mediators (eg. IL-6). We therefore set out to isolate the exosome fractions of rWNT5A stimulated Mewo cells and compared this to Mewo cells stimulated with carrier or rWNT3A as control (all stimulations 3 h). First of all, we could show that the isolated exosome fractions did contain exosomes by using electronmicroscopy (Figure 6A). Next, we showed that although the protein GAPDH was not increased upon rWNT5A stimulation (Figure 6B upper), the exosome related protein CD63 was (Figure 6B center). Also the exosome related Rab-protein Rab5b, was increased in the WNT5A stimulated fractions (Figure 6B lower). We could finally show that IL-6 and MMP2 were present in the rWNT5A stimulated exosome fractions as measured by Elisa of frozen exosome samples (Figure 6D-E and Additional file 1: Figure S3 C), while exosome-depleted supernatants from rWNT5A stimulated Mewo cells did not show elevated levels of IL-6 after freeze/thawing (Additional file 1: Figure S3 D). We also performed a microRNA (miRNA) microarray on the exosome fractions from rWNT5A stimulated Mewo cells (3 h) as compared to carrier stimulated Mewo cells. Although these data should be interpreted with caution due to low amounts of microRNA, elevated levels of four microRNAs were significantly increased in the rWNT5A induced exosomes (ENSG00000202498, hsa-mir-455, ENSG00000252531 and hsa-mir-593), as shown in Additional file 1 Table S1. Two of these miRNAs have previously been related to various forms of cancer (hsa-mir-455 [39] hsa-mir-593 [40]).

The WNT5A induced exosome release was dependent on Cdc42 as shown by transfecting Mewo cells with a DN-Cdc42, were the WNT5A induced IL-6 levels were decreased in exosomes specifically (Additional file 1: Figure S3 E) and not only in the supernatant as previously shown (Figure 3F). This was strengthened by the fact that a constitutively active form of Cdc42 (CA-Cdc42) expressed in Mewo cells induced exosome release on its own as measured by a quantitative Exosome ELISA (ExoELISA) (Figure 6C). The WNT5A induced exosome release was rapid with a maximum level at 3 h post WNT5A stimulation but also sustained over a long time period, measured up to 48 h post stimulation (data not shown). Interestingly, also the WNT3A stimulated control cells showed a marked increase in exosomal proteins (Figure 6B) but not the soluble mediators IL-6 and MMP2 (Figure 6D-E).

### WNT5A siRNA decreases endothelial cell co-culture branches

**Figure 7:**
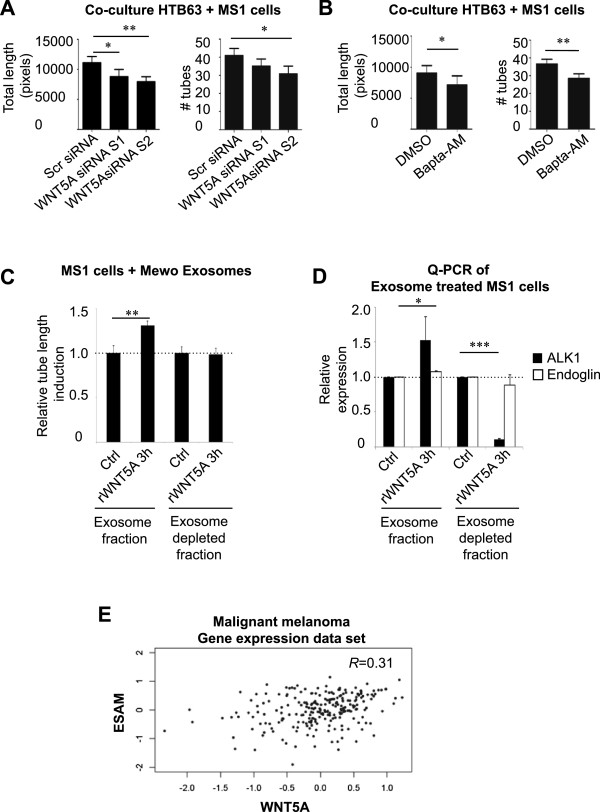
**WNT5A affects angiogenic processes in malignant melanoma cells. (A)** Quantification of total length of tubes (left), quantification of number of tubes (right), n = 4. Error bars represent SEM. * = p <0.05, ** = p <0.01 **(B)** MS1 cells grown together with HTB63 treated with DMSO or Bapta-AM, (left), total length of tubes, (right), number of tubes, n = 3. Error bars represent SEM. * = p <0.05 ** = p <0.01 by paired Student’s *t*-test. **(C)** MS1 cells that had been stimulated with exosomes isolated from carrier- or rWNT5A-treated Mewo cells, or with exosome-depleted supernatant from the corresponding samples, to analyze the relative total tube length induction, number of tubes, n = 6. Error bars represent SEM. ** = p <0.01 by paired Student’s *t*-test. **(D)** Q-PCR of mRNA from MS1 cells that had been stimulated with exosomes isolated from carrier- or rWNT5A-treated Mewo cells to analyze angiogenesis related genes using mouse specific primers. Both ALK1 (black bars) and Endoglin (white bars) expression was enhanced by rWNT5A induced exosomes (exosome fraction; left) whereas the exosome depleted supernatant fraction showed a loss of function as compared to total supernatant (exosome depleted fraction; right) **(E)** Correlation between WNT5A and the angiogenesis marker endothelial cell-selective adhesion molecule (ESAM) mRNA expression levels in a set of 223 primary malignant melanomas [24]. R = 0.31; Spearman’s rank test *P* = 7.7 × 10^-7^.

We next wanted to analyze whether the decreased levels of secreted mediators following WNT5A knockdown could have a functional implication on angiogenesis. Therefore, branching assays were performed using the mouse endothelial cell line MS1 in co-cultures with HTB63 melanoma cells transfected with WNT5A siRNA and subsequently seeded on a Matrigel layer. MS1 endothelial cells form tubular networks when cultured in Matrigel together with tumor cells. There was a decrease in the total length and in the number of tubes formed when MS1 cells were cultured together with cells transfected with WNT5A siRNA compared to cells transfected with Scrambled siRNA (Figure 7A). We also performed control cultures with MS1 cells only treated with rWNT5A in order to ensure that WNT5A in itself did not affect tube formation in the co-cultures (Additional file 1: Figure S3 F). We then used HTB63 cells that were pre-treated with Bapta-AM for 30 min before they were used in co-culture experiments (Figure 7B). There was a decrease in the total length and number of tubes formed after Ca^2+^ chelation compared to cells incubated only with vehicle (DMSO).

To verify that rWNT5A induced Mewo exosomes could signal to MS1 cells and that this affected angiogenesis related biological events we next examined the tube network formation of MS1 cells stimulated with isolated exosomes from untreated or rWNT5A treated Mewo cells (Figure 7C) or performed Q-PCR of MS1 cells stimulated with isolated exosomes from untreated or rWNT5A treated Mewo cells (3 h chosen as optimal time point) and analyzed induced expression of angiogenesis related genes (Figure 7D). We found that exosomes from rWNT5A treated Mewo cells induced MS1 tube formation (Figure 7C) while exosome-depleted supernatant from corresponding samples did not (Figure 7C). Similarly, exosomes from rWNT5A treated Mewo cells induced an increased expression of both ALK1 and endoglin in MS1 cells using mouse-specific primers, an effect that was abolished when using exosome-depleted supernatants as a control (Figure 7D).

To analyze whether WNT5A expression in malignant melanomas correlated to any known angiogenesis marker we performed a gene expression data set correlation using gene expression data from 223 primary malignant melanomas in Harbst et al. [24] (Figure 7E). WNT5A mRNA levels were found to correlate positively with the angiogenesis marker endothelial cell-selective adhesion molecule (ESAM) mRNA expression levels (R = 0.31; *P* = 7.7x10^-7^). Since the mRNA is originally prepared from paraffin samples the correlation can be viewed as a relatively strong.

## Discussion

Previous studies have correlated high WNT5A expression to poor prognosis in melanoma patients [11]. Angiogenesis and expression of immunomodulatory cytokines also correlate to poor prognosis in melanoma patients [8]. Here we show that, in melanoma cells with low endogenous WNT5A expression, the stimulation with rWNT5A induces a rapid release of exosomes containing the immunomodulatory cytokine IL-6 and the pro-angiogenic factors IL-8, VEGF and MMP2. We also show that, when cells with a high endogenous WNT5A expression were depleted of WNT5A, the release was reduced, suggesting that WNT5A is functioning in an auto- or paracrine manner. These changes were not accompanied by a change in mRNA expression excluding a possible transcriptional induction of these genes upon WNT5A signaling. We instead show that the WNT5A-induced effects on the release of these pro-angiogenic and immunomodulatory factors, are due to a WNT5A/Ca^2+^-regulated release of exosomes containing these mediators. In good agreement with this, the inhibitor of cAMP dependent Kinase (PKA), previously implicated in cytokine exocytosis [41,42], also inhibited the WNT5A-induced release of exosomes.

Changes in calcium signaling have been shown to induce a change in F-actin organization that can allow for secretory granules to reach the cell membrane and activate secretion [43]. We and others have previously shown that WNT5A induces an intracellular calcium signal in melanoma cells [22,44] and breast cells [5]. Also in this study, the Ca^2+^-chelator Bapta, inhibited the effects of WNT5A. Reorganization of F-actin from the prominent cell cortex towards the cytoplasmic region has previously been reported to be part of the Ca^2+^-dependent exocytosis mechanisms [14]. This has lately been correlated to activation of the small RhoGTPases Cdc42 and Rac1 [14,16,45] although the RhoGTPases probably mediate the vesicular trafficking primarily [14]. WNT5A was also shown to activate both Cdc42 and to a lesser extent Rac1 in breast cells [5]. Indeed, we could show that WNT5A induced activation of Cdc42 also in malignant melanoma Mewo cells and introduction of DN-Cdc42 and -Rac1 inhibited the WNT5A induced release.

Exosomes are produced by a wide range of mammalian cells and contribute to intercellular communications [18,19]. The exosomes may contain various molecules, also soluble mediators such as cytokines (eg. TGFβ and PGE2) [19,37], chemokines [46] and angiogenic factors (eg. VEGF and MMP2) [47]. It is known that exosomes act immunomodulatory on myeloid cells [18,48]. In immune cells, IL-6 and IL-8 mRNA expression has previously been connected to exosome signaling. Due to the fact that IL-6 mRNA levels remained unaffected in our study we believe that the mechanism behind WNT5A induced IL-6 release in malignant melanoma cells is Myd88/TLR2 independent [38,49]. Indeed, TLR2 was not expressed in the cell lines used in this study. We recently showed that also rWNT5A possess immunomodulatory effects on human monocytes. The findings in this study might explain why we observed a WNT5A specific induction of IL-6 and IL-10 mRNA in primary human monocytes that express TLRs at high levels specifically [27]. We now show, that in malignant melanoma cells, the induction of immunomodulatory and pro-angiogenic mediators are not caused by transcriptional activation, but by exosome-release of already formed proteins. Tumor cells generally have an increased exosome secretion that has been linked to angiogenesis, metastatic spread and immunosuppression [18]. A previous study even showed that the tumor microenvironment was able to specifically promote sorting of immunosuppressive factors into exosomes [37].

Exosome secretion is dependent on cytoskeletal reorganization and although it has previously been shown that the exosome dependent protein Rab35, can mediate the transport of Cdc42 to the plasma membrane to remodel the actin-structures [21], Cdc42 itself has not been connected to exosome release in mammals. In yeast however, Sec4p a Rab5b related protein, was shown to interact with Cdc42 to induce exocytosis [50]. Just as in the WNT5A induced malignant melanoma exosomes, Rab5b was expressed in plasma-derived exosomes from malignant melanoma patients [51]. Also, Rab5b was recently shown to participate in exosome-formation in malignant melanoma cells [20]. We propose that the mechanism behind the WNT5A induced exosome-release is Ca^2+^- and small RhoGTPase-regulated, affecting downstream proteins such as Rab5b and related Rab-family proteins [18-20,51]. It should not be excluded that also other Ca^2+^-regulated proteins, affecting cortical F-actin disassembly, could affect the exosome release [52,53]. In this study, even the canonical WNT3A protein induced an increase in exosome-release. We suggest that an independent signaling pathway, distinct from the Ca^2+^-induced, non-canonical WNT5A-pathway, causes this release. Strengthening this hypothesis, the exosomes produced by canonical WNT3A displayed a different content as compared to those produced by non-canonical WNT5A signaling. Or it could be explained by the recent finding that indeed Wnt proteins (WNT3A and WNT5A) are secreted on exosomes [54,55]. It has long been known in the Wnt field that WNT5A can induce expression of it self. The mechanism behind has not been explained and the data in this study together with the mentioned studies might partly explain how this loop could work.

Although numerous articles describing the effects of WNT5A on intracellular signaling proteins have been published, few studies concerning WNT5A and transcriptional regulation are available. We also show that WNT5A does not affect the factors analyzed in this study at the transcriptional level.

## Conclusion

In the present study we present novel data showing that the non-canonical Wnt protein WNT5A, has an effect on release of exosomes containing immunomodulatory and pro-angiogenic factors in malignant melanoma cells. This might have an impact on angiogenic processes in malignant melanomas as supported by our finding that indeed the expression of WNT5A correlates to that of the angiogenesis associated gene ESAM in a gene expression data set correlation of 223 malignant melanomas, and also has a biological effect on *in vitro* branching experiments using endothelial co-cultures. We suggest that, by affecting the exocytosis of numerous secreted factors in sensitive systems such as the cell rich microenvironment of a tumor, WNT5A signaling can have a much wider biological consequence than has previously been described.

## Methods

### Cell culture and treatments

All malignant melanoma cell lines were purchased from the American Type Tissue Collection (ATCC, Manassas, VA). MS1 murine endothelial cells [56] were a gift from Professor Kristian Pietras, (Lund University, Sweden). Recombinant proteins (rWNT5A (0.2 μg/ml) or rWNT3A (0.05 μg/ml) were from R&D systems, Minneapolis, MN. All chemicals and inhibitors were purchased from Sigma Aldrich (St Louis, MO.) unless otherwise noted and used at concentrations: 10 μM Bapta-AM or DMSO control, 5 μM PKA inhibitor H89, 10 ng/ml Tetanus Toxin or 10 μg/ml Brefeldin A. The antibodies used were Goat anti-WNT5A (R&D systems, Minneapolis, MN), anti-CD63, anti-Rab5B and anti-GAPDH (Santa Cruz biotechnologies), mouse anti-β-actin (MP biomedicals). For BD Cytometric Bead Array (CBA) the Human Inflammation Kit was used (BD Biosciences). For hematoxylin and eosin cells were fixed in 4% PFA and embedded in paraffin and then stained. F-actin cytoskeleton was stained using Alexafluor546-coupled Phalliodin. For ELISAs Quantikine Human IL-6 ELISA kit, Quantikine Human MMP2 ELISA kit and Quantikine Human VEGF ELISA kit from R&D systems (Minneapolis, MN) was used. Exosome Elisa (ExoELISA) CD63 ExoELISA^TM^ from System Biosciences (Uden, The Netherlands) was used.

### RNA extraction and RT-QPCR

mRNA expression was analyzed using RT-QPCR. RNA was extracted using RNeasy kit (Qiagen, Hilden, Germany). For primer sequences, see Additional file 1: Figure S4 and [57].

### Transfection and knockdown experiments using siRNA

For overexpression of CA-Cdc42, DN-Cdc42 and Rac1 (N17), Mewo cells were transfected using 1 μg plasmid (a kind gift from Dr Pontus Aspenström, (Karolinska Institutet, Sweden) per 24-well. 2 μg CA-Cdc42, DN-Cdc42 or pcDNA3.1 was used per 6-well for ExoELISA experiments. For knockdown experiments 150000 HTB63 cells were transfected with Silencer Select siRNA against WNT5A (10nM, Applied Biosystems Carlsbad CA) and the appropriate concentrations of scrambled siRNA as control.

### MS1 Co-culture experiments

Co-culture experiments were performed as previously published [58]. For exosome stimulation experiments, MS1 cells were seeded in gelatine-coated 12-well plates and stimulated with exosome-enriched or –depleted fractions from carrier or rWNT5A (0.3 μg/ml, 3 h) stimulated Mewo cells (see below). The primers used for angiogenesis specific Q-PCR of the MS1 cells have been published previously [57].

### Exosome isolation

Mewo cells were cultured over night in serum free Eagle’s Minimum Essential Medium supplemented with penicillin/streptomycin that had been Ultracentrifuged for 2 h at 100,000 g using Beckman Optima TLX Ultracentrifuge. The medium was replaced by fresh Ultracentrifuged medium and cells were stimulated with carrier, rWNT3A or rWNT5A as indicated for 3 h. The supernatants were collected and exosome isolation was carried out by differential centrifugation. Briefly, the supernatants were centrifuged for 300 g for 10 min to remove debris. The supernatants were then centrifuged once at 2,000 g for 15 min, once at 10,000 g for 30 min and finally exosomes were pelleted at 100,000 g for 1,5-2 h.

### Exosome identification by electron microscopy

The exosome enriched pellet was diluted in 50 μl PBS and kept at 4°C until EM analysis. A drop of the exosome sample was placed on a carbon coated copper grid and was let to adhere for 1 min. The sample was contrast stained by adding a drop of 2% uranyl acetate to the sample on the grid. Excess liquid was removed by gently using an absorbing paper, before positioning the grid on a paper with the coated side up and was let to air dry for 5 minutes. The preparation was examined using an electron microscope FEI Tecnai spirit at 100 KV.

### microRNA (miRNA) expression analysis

MicroRNA expression analyses were performed using Affymetrix miRNA-3_0 arrays according to the manufacturer’s instructions (Affymetrix, Santa Clara, CA). These array experiments were performed at Swegene Centre for Integrative Biology (SCIBLU) at Lund University. Microarray data were initially pre-processed and normalized using Robust Multi-array Analysis (RMA) method [59]. These analyses were performed using Affymetrix Expression Console Software v1.1.2. To identify significantly differentially expressed miRNAs between carrier stimulated and rWNT5A stimulated Mewo-exosomes, we used significance analysis of microarrays (SAM) method [60]. SAM analyses were performed using TMEV v4.0 software.

### Statistical analysis

All data was analyzed using Graphpad Prism software and is visualized as mean with error bars representing standard deviation (SD) or standard error of the mean (SEM). Statistical significance was calculated using ANOVA or Student’s *t*-test as indicated in the Figure legends. Spearman’s rank test was used for the statistical analysis of the gene expression data set. The malignant melanoma mRNA data set has previously been published [24] and approved by the local ethics committee of Lund University (Dnr 191/2007). The mRNA was prepared from paraffin embedded samples of 223 primary malignant melanomas.

## Abbreviations

MMP: Matrix metalloproteinase; SNARE: Synaptic soluble NSF attachment protein receptors; SNAP: Soluble NSF attachment proteins; VAMPs: Vesicle-associated membrane proteins; TeNT: Tetanus neurotoxin; TIMP: Tissue inhibitor or metalloproteinases; CBA: Cytometric bead array; DN: Dominant negative.

## Competing interests

T.A. is a shareholder of WntResearch and is also part-time CSO of the company. The other authors declare no conflict of interest.

## Authors’ contributions

EJE performed the majority of the experiments, designed experiments and wrote the manuscript together with KL and TA, CB, VB, and FS performed experiments. EC performed the electron microscopy. GJ was responsible for the microarray analyses. KL was responsible for designing the study and writing the final manuscript. All authors read and approved the final manuscript.

## Supplementary Material

Additional file 1: Table S1 miRNA microarray of RNA isolated from Mewo exosomes stimulated or not with rWNT5A for 3h. **Figure S1.***IL-6 and VEGF is increased in A375 cell* culture supernatants after Wnt5a treatment (A). (B) Corresponding RT-QPCR for IL-6. (C-D) As in (A-B) but measuring IL-6 using A2058 cells. (E) Mewo cells treated with PMA/ionomycin (12h) as positive control for RT-QPCR (IL-6). **Figure S2.** (A) Mewo cells treated with rWNT5A for 3h stained with phalloidin to visualize F-actin. (B and D) Transfection of Mewo cells with DN-Cdc42, but not -Rac1, inhibits the Wnt5a induced secretion of VEGF (B) or MMP (D). The control cells were transfected with an empty vector. (C and D) Mewo cells were pre-treated with Brefeldin A for 30min prior to Wnt5a treatment for 3h. **Figure S3.** (A) IL-6, VEGF and MMP2 levels (actual concentration) as measured by Elisa of non-frozen or freeze-thawed cell culture supernatants of Mewo cells that were treated with rWNT5A or carrier (3h) n = 2. (B) Western blot of TLR2 in Mo-mDCs (Ctrl), A375, Mewo and HTB63 cells. (C) IL-6 or MMP2 levels (actual concentration) as measured by Elisa of frozen exosome fractions isolated from supernatant of Mewo cells that were treated with 0.2μg/ml rWNT5A, carrier or 0.1ng/ml rWnt3a for 3h. (D) Induction of IL-6 levels in unfractionated supernatants of rWNT5A stimulated Mewo cells (ratio; black bars) but not in exosome-depleted supernatants of rWNT5A stimulated Mewo cells (ratio; white bars). (E) IL-6 levels in frozen exosome fractions isolated from supernatant of transfected and treated Mewo cells as indicated. (F) MS1 cells grown alone and treated with carrier or rWNT5A, total length of tubes (left), number of tubes (right). **Figure S4.** Table of primers used for Q-PCR. * = p <0.05; ** = p <0.01; *** = p <0.001 by Student’s *t*-test. n=3-7 unless otherwise stated. Click here for file
