# Supplementary material for: WNT5A induces release of exosomes containing pro-angiogenic and immunosuppressive factors from malignant melanoma cells
Source: Mol Cancer. 2014 Apr 26;13:88. doi: 10.1186/1476-4598-13-88 (PMC4022450; doi:10.1186/1476-4598-13-88)
Supplement: Additional file 1: Table S1 — miRNA microarray of RNA isolated from Mewo exosomes stimulated or not with rWNT5A for 3h. Figure S1.IL-6 and VEGF is increased in A375 cell culture supernatants after Wnt5a treatment (A). (B) Corresponding RT-QPCR for IL-6. (C-D) As in (A-B) but measuring IL-6 using A2058 cells. (E) Mewo cells treated with PMA/ionomycin (12h) as positive control for RT-QPCR (IL-6). Figure S2. (A) Mewo cells treated with rWNT5A for 3h stained with phalloidin to visualize F-actin. (B and D) Transfection of Mewo cells with DN-Cdc42, but not -Rac1, inhibits the Wnt5a induced secretion of VEGF (B) or MMP (D). The control cells were transfected with an empty vector. (C and D) Mewo cells were pre-treated with Brefeldin A for 30min prior to Wnt5a treatment for 3h. Figure S3. (A) IL-6, VEGF and MMP2 levels (actual concentration) as measured by Elisa of non-frozen or freeze-thawed cell culture supernatants of Mewo cells that were treated with rWNT5A or carrier (3h) n = 2. (B) Western blot of TLR2 in Mo-mDCs (Ctrl), A375, Mewo and HTB63 cells. (C) IL-6 or MMP2 levels (actual concentration) as measured by Elisa of frozen exosome fractions isolated from supernatant of Mewo cells that were treated with 0.2μg/ml rWNT5A, carrier or 0.1ng/ml rWnt3a for 3h. (D) Induction of IL-6 levels in unfractionated supernatants of rWNT5A stimulated Mewo cells (ratio; black bars) but not in exosome-depleted supernatants of rWNT5A stimulated Mewo cells (ratio; white bars). (E) IL-6 levels in frozen exosome fractions isolated from supernatant of transfected and treated Mewo cells as indicated. (F) MS1 cells grown alone and treated with carrier or rWNT5A, total length of tubes (left), number of tubes (right). Figure S4. Table of primers used for Q-PCR. * = p <0.05; ** = p <0.01; *** = p <0.001 by Student’s t-test. n=3-7 unless otherwise stated. [file 1476-4598-13-88-S1.pdf]

Table S1. miRNA microarray of RNA isolated from Mewo exosomes stimulated or not with rWNT5A for 3h. The values show the relative increase (fold change) of miRNA in the rWNT5A stimulated exosomes as compared to carrier stimulated.

Probe Set ID	Sequence type	Fold change	q-value	Alternative name
**ENSG00000202498**	snoRNA	8.252258	0	SNORD116
**hsa-mir-455**	miRNA	2.4449663	0	
**ENSG00000252531**	snoRNA	2.222854	0	SNORA73
**hsa-mir-593**	Stem-loop	1.8366694	0	

Figure S1

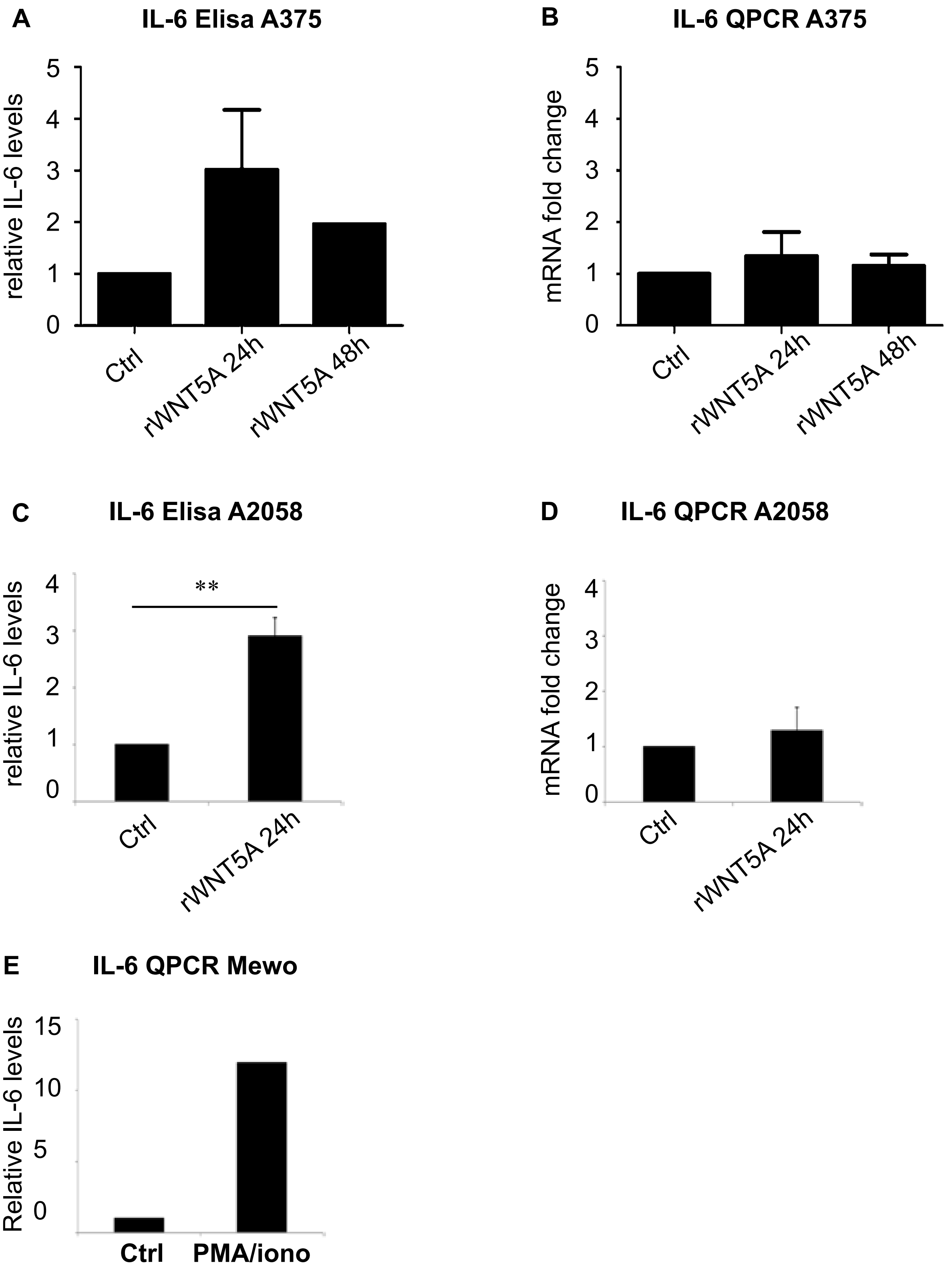
A
IL-6 Elisa A375
relative IL-6 levels
5
4
3
2
1
0
Ctrl
rWNT5A 24h
rWNT5A 48h
B
IL-6 QPCR A375
mRNA fold change
5
4
3
2
1
0
Ctrl
rWNT5A 24h
rWNT5A 48h
C
IL-6 Elisa A2058
relative IL-6 levels
4
3
2
1
0
**
Ctrl
rWNT5A 24h
D
IL-6 QPCR A2058
mRNA fold change
4
3
2
1
0
Ctrl
rWNT5A 24h
E
IL-6 QPCR Mewo
Relative IL-6 levels
15
10
5
0
Ctrl
PMA/iono

Figure S2

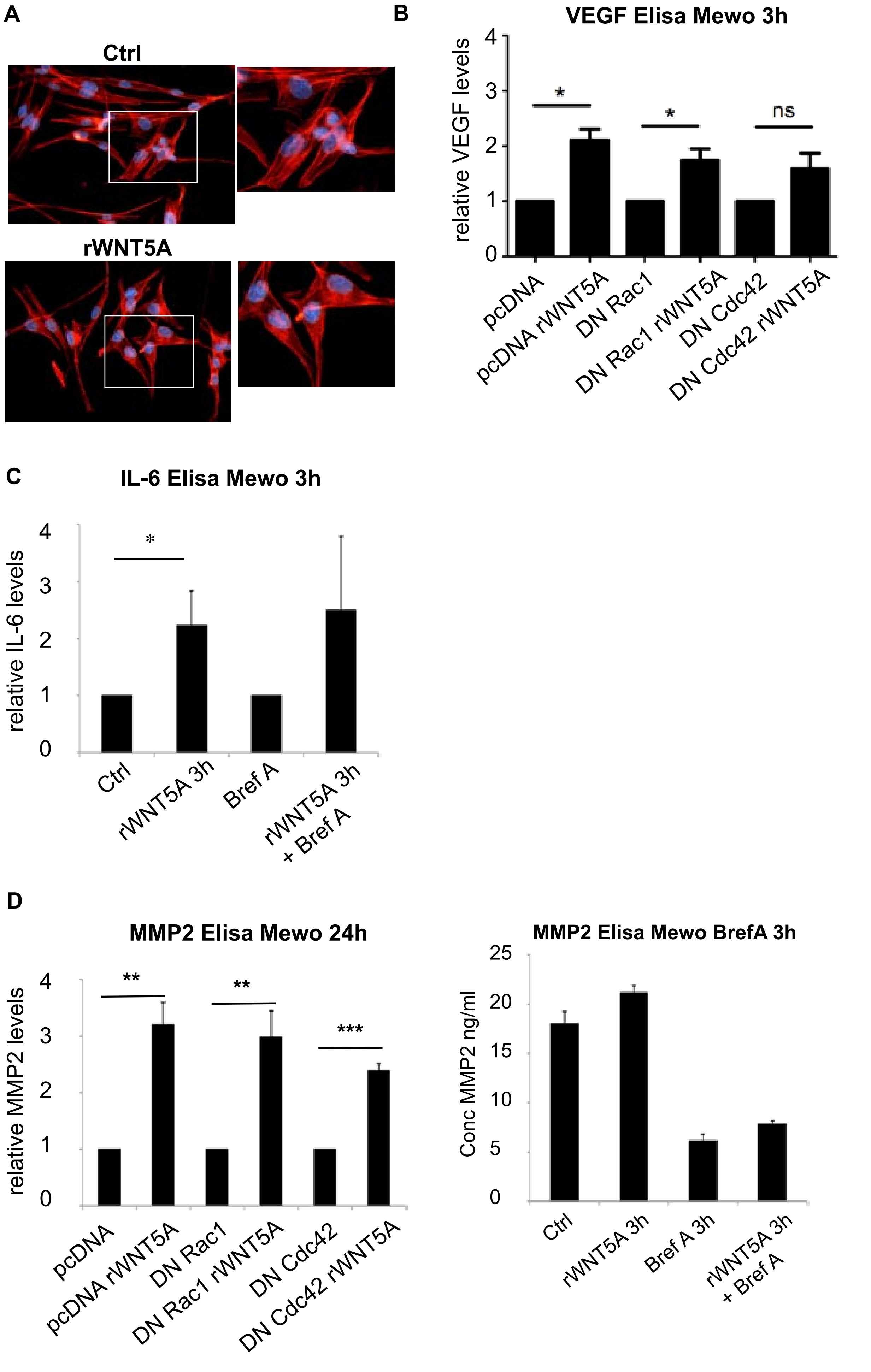

A
Ctrl
rWNT5A
B
VEGF Elisa Mewo 3h
relative VEGF levels
0
1
2
3
4
*
*
ns
pcDNA
pcDNA rWNT5A
DN Rac1
DN Rac1 rWNT5A
DN Cdc42
DN Cdc42 rWNT5A
C
IL-6 Elisa Mewo 3h
relative IL-6 levels
0
1
2
3
4
*
Ctrl
rWNT5A 3h
Bref A
rWNT5A 3h + Bref A
D
MMP2 Elisa Mewo 24h
relative MMP2 levels
0
1
2
3
4
**
**
***
pcDNA
pcDNA rWNT5A
DN Rac1
DN Rac1 rWNT5A
DN Cdc42
DN Cdc42 rWNT5A
MMP2 Elisa Mewo BrefA 3h
Conc MMP2 ng/ml
0
5
10
15
20
25
Ctrl
rWNT5A 3h
Bref A 3h
rWNT5A 3h + Bref A

Figure S3

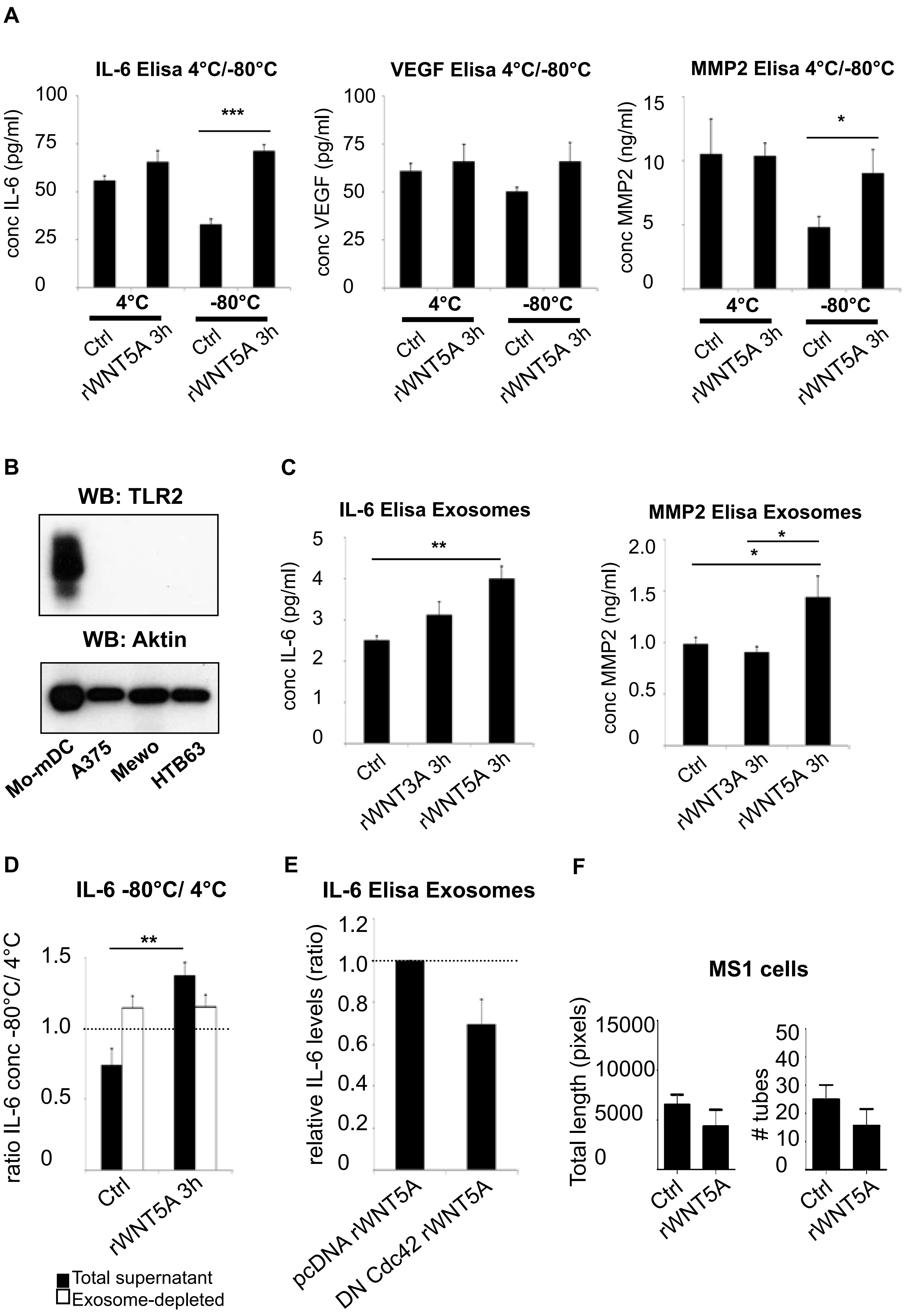
A
IL-6 Elisa 4°C/-80°C
conc IL-6 (pg/ml)
100
75
50
25
0
***
4°C
-80°C
Ctrl
rWNT5A 3h
Ctrl
rWNT5A 3h
VEGF Elisa 4°C/-80°C
conc VEGF (pg/ml)
MMP2 Elisa 4°C/-80°C
conc MMP2 (ng/ml)
15
10
5
*
B
WB: TLR2
WB: Aktin
Mo-mDC
A375
Mewo
HTB63
C
IL-6 Elisa Exosomes
conc IL-6 (pg/ml)
**
rWNT3A 3h
MMP2 Elisa Exosomes
conc MMP2 (ng/ml)
2.0
1.5
1.0
0.5
D
IL-6 -80°C/ 4°C
ratio IL-6 conc -80°C/ 4°C
Total supernatant
Exosome-depleted
E
IL-6 Elisa Exosomes
relative IL-6 levels (ratio)
1.2
1.0
0.8
0.6
0.4
0.2
pcDNA rWNT5A
DN Cdc42 rWNT5A
F
MS1 cells
Total length (pixels)
15000
10000
5000
tubes
50
40
30
20
10
rWNT5A

Supplementary Fig 4

Gene	Forward Sequence 5'-3'	Reverse Sequence 5'-3'
IL-6	GGCACTGGCAGAAAACAACC	GCAAGTCTCCTCATTGAATCC
Wnt5a	TCAGGACCACATGCAGTA	CTCATGGCGTTCACCACC
VEGF	AGGAGGAGGGCAGAATCATCA	CTCGATTGGATGGCAGTAGCT
UBC	ATTTGGGTCGCGGTTCTTG	TGCCTTGACATTCTCGATGGT
SDHA	TGGGAACAAGAGGGCATCTG	CCACCACTGCATCAAATTCATG
YWHAZ	ACTTTTGGTACATTGTGGCTTCAA	CCGCCAGGACAAACCAGTAT
MMP2	TGA TCT TGA CCA GAA TAC CAT CGA	GGC TTG CGA GGG AAG AAG TT

Supplementary Figure Legends

Figure S1

IL-6 and VEGF is increased in A375 cell culture supernatants after Wnt5a treatment.

(A) A375 cells treated with rWNT5A 0.2μg/ml for 24h and 48h. Elisa was used to measure differences in IL-6 (A). IL-6 n=2. Error bars represent SEM. (B) Changes in mRNA were analyzed using RT-QPCR for IL-6. At least 4 experiments were performed (n=4-7). Error bars represent SEM. (C-D) A2058 cells treated with rWNT5A 0.2μg/ml for 24h. Elisa was used to measure differences in IL-6 (C). IL-6 n=3. Error bars represent SD. ** $p<0.01$ Students t-test. (D) Changes in mRNA were analyzed using RT-QPCR for IL-6. n=4. Error bars represent SD. (E) Mewo cells treated with PMA/ionomycin for 12h was used as positive control for RT-QPCR (IL-6 shown in E).

Figure S2. (A) Mewo cells treated with rWNT5A for 3h stained with phalloidin to visualize F-actin and counter stained with DAPI. The white boxes represent magnified areas. (B) Transfection of Mewo cells with DN-Cdc42, but not -Rac1, inhibits the Wnt5a induced secretion of VEGF as measured by Elisa. The control cells were transfected with an empty vector. The data shown are ratios of rWNT5A treated (3h)/ untreated cells for each plasmid transfected and normalized against the empty vector control. n=4. * = $p <0.05$ by paired Student's t-test. Error bars represent SEM. (C) Mewo cells were pre-treated with Brefeldin A, Bref A, for 30min prior to Wnt5a treatment for 3h. IL-6 levels in cell-culture supernatants were detected using Elisa. n=3. *= $p <0.05$ by Student's t-test. Error bars represent SD. (D) Left panel; Transfection of Mewo cells with DN-Cdc42 inhibits the Wnt5a induced secretion of MMP2 as measured by Elisa. DN-

Rac1 does not. The control cells were transfected with an empty vector. The data shown are ratios of rWNT5A treated (3h)/ untreated cells for each plasmid transfected and normalized against the empty vector control. n=3. Right panel; Mewo cells were pre-treated with Brefeldin A, Bref A, for 30min prior to Wnt5a treatment for 3h. MMP2 levels in cell-culture (actual concentration) supernatants were detected using Elisa n=4. Error bars represent SEM.

Figure S3. (A) IL-6, VEGF and MMP2 levels as measured by Elisa in cell culture supernatants of Mewo cells that were treated with 0.2μg/ml rWNT5A or carrier for 3h and subsequently was freshly run on Elisa (4°C) or freeze/thawed for two cycles -80°C/4°C before analyses. The bar graphs show actual concentration for each treatment indicated. Error bars represent SD. n= 2. (B) Western blot of TLR2 in Mo-mDCs (Ctrl), A375, Mewo and HTB63 malignant melanoma cells. (C) IL-6 or MMP2 levels as measured by Elisa of frozen exosome fractions isolated from supernatant of Mewo cells that were treated with 0.2μg/ml rWNT5A, carrier or 0.1ng/ml rWnt3a for 3h. The bar graphs show the actual concentration for each treatment indicated. Error bars represent SD. n= 3. * = $p < 0.05$; **= $p < 0.01$; ***= $p < 0.001$ by Student's t-test. (D) Induction of IL-6 levels in unfractionated supernatants of rWNT5A stimulated Mewo cells, as measured by Elisa of frozen/non-frozen supernatants (ratio; black bars) but not in exosome-depleted supernatants of rWNT5A stimulated Mewo cells, as measured by Elisa of frozen/non-frozen exosome-depleted supernatants (ratio; white bars). The bar graphs show the relative increase of IL-6 in frozen : refrigerated samples. SEM. n=3. **= $p < 0.01$ by Student's t-test. (E) IL-6 levels, as measured by Elisa, of frozen exosome fractions isolated from supernatant of Mewo cells that were transfected with pcDNA3 or DN-Cdc42 plasmids and subsequently treated with carrier or 0.2μg/ml rWNT5A for 3h. The bar graphs show the relative increase of rWNT5A treated as

compared to carrier treated, for each transfection indicated. Error bars represent SEM. n=4. (F) MS1 cells grown alone and treated with carrier or rWNT5A, total length of tubes (left), number of tubes (right), n=3. Error bars represent SEM.

Figure S4,

Table of primers used for Q-PCR.
